# Data-driven simulations to assess the impact of study imperfections in time-to-event analyses

**DOI:** 10.1093/aje/kwae058

**Published:** 2025-01-08

**Authors:** Michal Abrahamowicz, Marie-Eve Beauchamp, Anne-Laure Boulesteix, Tim P. Morris, Willi Sauerbrei, Jay S. Kaufman

**Affiliations:** 1 Department of Epidemiology, Biostatistics and Occupational Health, McGill University, Montreal, QC H3A 1Y7, Canada; 2 Centre for Outcomes Research and Evaluation (CORE), Research Institute of the McGill University Health Centre, Montreal, QC H4A 3S5, Canada; 3 Institute for Medical Information Processing, Biometry, and Epidemiology, Faculty of Medicine, Ludwig-Maximilians-Universität München, 81377 Munich, Germany; 4 Munich Center of Machine Learning, Munich, Germany; 5 MRC Clinical Trials Unit at UCL, Institute of Clinical Trials & Methodology, University College London, London WC1V 6LJ, United Kingdom; 6 Institute of Medical Biometry and Statistics, Faculty of Medicine and Medical Center, University of Freiburg, 79104 Freiburg, Germany

**Keywords:** simulations, quantitative bias analysis, survival analysis, sensitivity analyses, noncollapsibility, time-varying exposure, interval censoring

## Abstract

Quantitative bias analysis (QBA) permits assessment of the expected impact of various imperfections of the available data on the results and conclusions of a particular real-world study. This article extends QBA methodology to multivariable time-to-event analyses with right-censored endpoints, possibly including time-varying exposures or covariates. The proposed approach employs data-driven simulations, which preserve important features of the data at hand while offering flexibility in controlling the parameters and assumptions that may affect the results. First, the steps required to perform data-driven simulations are described, and then two examples of real-world time-to-event analyses illustrate their implementation and the insights they may offer. The first example focuses on the omission of an important time-invariant predictor of the outcome in a prognostic study of cancer mortality, and permits separating the expected impact of confounding bias from noncollapsibility. The second example assesses how imprecise timing of an interval-censored event—ascertained only at sparse times of clinic visits—affects its estimated association with a time-varying drug exposure. The simulation results also provide a basis for comparing the performance of two alternative strategies for imputing the unknown event times in this setting. The R scripts that permit the reproduction of our examples are provided.

## Introduction

Most epidemiologic studies face some imperfections of the available data and limitations of the study design that may affect the accuracy of results and sometimes invalidate the conclusions. Examples include unmeasured confounders,^1–3^ measurement errors,^4,5^ selection bias^6–8^ or sparse measurements of time-varying predictors.^9,10^

Instead of just recognizing such imperfections in the discussion section of the published papers, recent studies increasingly employ quantitative bias analysis (QBA) for a more formal assessment of their impact on results.^11–15^ The traditional QBA approach relies on substantive knowledge and literature to derive quantitative assumptions regarding relevant bias parameters, in order to repeatedly correct observed data through multiple probabilistic imputation of, eg, an unmeasured confounder or measurement errors.^11,12^ These sophisticated sensitivity analyses allow researchers to assess how the estimate of interest may change if the relevant imperfection was avoided. QBA methods have been developed for a wide range of settings, including time-to-event analyses where they permit, eg, assessment of the joint impact of different data imperfections^16^ or correction for exposure measurement errors in a binary time-varying exposure.^17^ However, a traditional probabilistic QBA approach does not allow quantifying the impact of the imperfection on bias, mean squared error, or coverage rate, quantifying the impact of the imperfection on bias, mean squared error, or coverage rate.^18^

We propose a simulation-based approach that complements traditional QBA methods, and may be especially useful in multi-variable time-to-event analyses, possibly with time-varying exposures (TVEs). In particular, our data-driven simulations permit directly assessing how the estimates obtained using imperfect data are expected to diverge from the true parameter value, while accounting for important features of the specific real-world dataset. Real-world applications are illustrated by two time-to-event analyses, one assessing the impact of an unmeasured prognostic factor and the other involving a TVE and an imprecisely timed event.

## Methods

### Proposed approach to data-driven simulations for time-to-event analysis

We propose to combine (1) observed multivariable real-world data with (2) simulating additional data items (eg, outcomes and/or covariates) based on carefully defined assumptions. Simulations permit assessing the impact of the imperfection on, for example, bias and coverage rate, whereas using real-world data ensures the relevance of results for a particular empirical study. Our data-driven simulation approach involves the following 7 steps. Practical implementation of these steps is later illustrated in two real-world examples, each dealing with a different data imperfection. Steps 1-3 are the preliminary steps. Steps 4-6 are to be repeated across *m* independent repetitions for each scenario identified in step 3. Step 7 is the final step.

**Step 1. Identification of data imperfections.** Identify the data imperfection(s) of interest in the available real-world dataset and, if relevant, conduct preliminary analyses to assess, eg, their frequency and/or patterns.
**Step 2. Initial analyses not corrected for the imperfections.** Fit the chosen regression models (usually multivariable) to get initial uncorrected estimates of the relationships of the exposure and available covariates with the outcome.
**Step 3. Simulation assumptions.** Formulate assumptions underlying data simulations, in two substeps:
3a. Specify a few plausible alternative parameter “true” values for the association of primary interest, including null and one close to the initial step 2 estimate.
3b. In addition, based on substantive knowledge, formulate assumption(s) regarding how available data can be modified or expanded to artificially create “oracle datasets,” free of the imperfection(s) identified in step 1. Several scenarios may involve different combinations of assumptions in 3a and 3b.
**Step 4. Oracle dataset generation.** Generate an oracle dataset, free of the imperfection(s) of interest, that combines the relevant empirical initial step 2 estimates with additional data simulated according to step 3b assumptions.
**Step 5. Imperfect dataset generation.** Modify the oracle dataset from step 4 to account for the relevant imperfection(s).
**Step 6. Analyses.** Analyze (6a) oracle and (6b) imperfect (modified) datasets (from steps 4 and 5, respectively), using the same methods.
**Step 7. Final step: summarizing results.** Summarize results of step 6 across the *m* repetitions, for a given scenario, using the performance characteristics of interest.^18^ Contrast the corresponding results for oracle versus imperfect data, to assess the expected impact of the data imperfection(s) on real-world initial results from step 2.

Both real-world examples, discussed below, employ *m* = 1000 repetitions, which ensures that the 95% CI for bias will exclude 0 with about 90% power if the bias corresponds to 10% of the empirical SE of estimates,^19^ implying a standardized difference of 10% between the mean estimate and true parameter value (see Appendix S1 for details). In other applications, the required number of repetitions may be calculated to achieve the desired precision for other performance characteristics listed, eg, in Morris et al.^18^

For time-to-event analyses, in step 4 we adapted the validated permutational algorithm^20–22^ to assign each event time observed in the real-world dataset to one of the individuals’ multivariable vectors of (observed or simulated) covariate/exposure values. To this end, for each event time *t*, we calculated the corresponding, possibly time-varying, hazard ratios *HR*_*i*_(*t*) for each individual *i* in the risk set, based on the true data-generating model, defined to reflect assumptions from step 3. The algorithm uses weighted random sampling, with probabilities proportional to *HR*_*i*_(*t*), to assign the event at time *t* to 1 individual, who is then removed from all future risk sets.^20–22^ Appendixes S3 and S4 describe the algorithm implementation for each example.

## Example 1: Assessing the impact of omitting an important prognostic factor

### Empirical research question and data source

To illustrate the impact of lacking data on an important prognostic factor in multivariable time-to-event analyses, we considered the association between colon obstruction by a tumor and all-cause mortality in colon cancer patients in a publicly available dataset from the R (R Foundation for Statistical Computing) survival package.^23,24^ Among *n* = 906 patients without missing data, 175 (19.3%) had the colon obstructed, and 441 died during follow-up. Some patients’ characteristics, assessed at diagnosis, were associated with colon obstruction and/or mortality, and some were correlated with each other (see Tables S1 and S2).

R code to reproduce our data-driven simulations is available at the link provided in the Data Availability statement.

**Figure 1:**
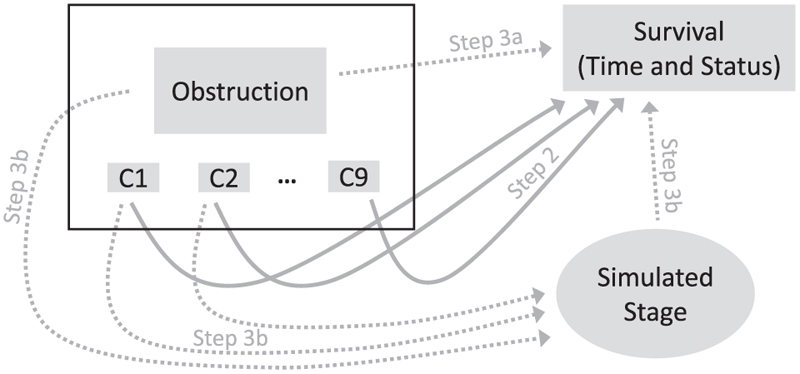
Illustration of how oracle datasets were created for Example 1, by combining real-word with simulated data. Rectangles represent data taken directly from the real-word dataset. In particular, individual data for obstruction and covariates (C1, …, C9), shown in the black rectangle on top left, were extracted without any modifications, implying that all relationships between these variables were preserved. Survival outcomes, consisting of outcome times and status (event or censoring), were extracted from the real-word dataset, but in different simulated oracle samples, each outcome was assigned to different subjects using the permutational algorithm. The oval represents the simulated data on cancer stage. Arrows identify relationships considered when either simulating cancer stage or assigning outcomes to individual study participants. For dotted arrows, the strength of the associations (hazard ratio or odds ratio) used for data generation was based on the corresponding assumptions from step 3. For solid arrows, the strength of associations used to simulate datasets was based on the adjusted HR for each covariate estimated in step 2 using the real-world data.

**Step 1. Identification of the data imperfection.** The available data do not include cancer stage at diagnosis,^23,24^ a powerful predictor of mortality in colorectal cancer patients,^25,26^ likely associated with colon obstruction.^27,28^
**Step 2. Initial analyses not corrected for the imperfection.** In a Cox proportional hazards (PH) model adjusting for measured covariates, colon obstruction was associated with a moderately increased all-cause mortality (hazard ratio [HR] of 1.33; 95% CI, 1.06-1.68; Table S1).
**Step 3. Simulation assumptions.**
3a. In 4 scenarios, we assumed a true HR for the colon obstruction-mortality association of 1.0, 1.3 (similar to initial step 2 estimate), 1.5, or 2.0.
3b. Based on clinical literature and discussions with a colorectal cancer epidemiologist, we assumed that higher III/IV cancer stage at diagnosis (1) had a prevalence of about 35%, (2) was associated with much higher mortality (HR = 4.0),^25,26^ and (3) was associated with colon obstruction^27,28^ (odds ratio [OR] of 1.2) and selected covariates (see Appendix S2). Additional scenarios assumed either a null (OR = 1.0) or strong (OR = 2.0) obstruction-stage association. Finally, to confirm that initial results can be replicated, we added a scenario with no obstruction-stage (OR = 1.0) and no stage-mortality (HR = 1.0) associations, and with obstruction-mortality HR = 1.3, similar to the initial estimate.
**Step 4. Oracle dataset generation.** The *m* = 1000 oracle datasets were generated by adapting the permutational algorithm,^20–22^ using 4 substeps (see Appendix S3 for more details). Substeps 4a-4b were common to all repetitions, and substeps 4c-4d were repeated independently for each repetition:
4a. Extract the 906 individual **X**_*i*_ vectors of observed values of colon obstruction exposure and all measured covariates, preserving all their relationships.
4b. Independently, extract all 906 observed real-world outcomes: 441 times of death and 465 censoring times.
4c. Generate a binary indicator of higher cancer stage *S*_*i*_ for each patient, *i* = 1,…,906, from the binomial distribution, with a probability calculated using a multivariable logistic model based on patient-specific **X**_*i*_ vectors from substep 4a and corresponding ORs assumed in step 3 (see Appendix S2 for details).
4d. Assign each outcome to 1 individual, *i* = 1,…,906, defined by the vector (**X**_*i*_, *S*_*i*_), using the “true” multivariable PH model with HRs for obstruction exposure and stage based on assumptions from step 3, and step 2 HR estimates for measured covariates.
Figure 1 shows how different elements of observed real-world (rectangles) and simulated (oval) data were combined to generate the oracle datasets. Solid arrows indicate relationships whose strength was based on initial step 2 estimates, while dotted arrows represent those based on assumptions from step 3.
**Step 5. Imperfect dataset generation.** For each oracle dataset generated at step 4, we deleted the simulated cancer stage but kept unchanged the values of exposure, measured covariates and the outcomes assigned by the permutational algorithm.
**Step 6. Analyses.** Each simulated dataset was analyzed independently using a multivariable Cox PH model that included the exposure and all measured covariates but either did (oracle datasets) or did not (imperfect datasets) adjust for the simulated cancer stage.
**Step 7. Final step: summarizing results.** Table 1 summarizes results for colon obstruction exposure from multivariable Cox PH models fitted to 1000 either oracle or imperfect datasets for each scenario. Oracle estimates have only minimal relative biases < 2.5% and coverage rates very close to the nominal 95%, which indirectly validates our simulation methods.

In contrast, for most simulated scenarios, the model fitted to imperfect data, without cancer stage, yielded moderately biased estimates, with the 95% CI for bias excluding 0 (indicated by footnote b in column 12 of Table 1). The overestimation vs. under-estimation of the true log(HR) in different scenarios reflects the double-edged impact of not adjusting for stage. In scenarios 1-4, advanced stage is both (weakly) associated with colon obstruction (OR = 1.2) and (strongly) associated with mortality (HR = 4.0). Thus, not adjusting for stage induces overestimating the obstruction HR, due to confounding. Yet omitting a strong predictor also shifts all estimates toward the null due to noncollapsibility of HRs.^29,30^ Thus, imperfect exposure estimates reflect a trade-off between confounding by stage versus noncollapsibility.^31–33^ This trade-off depends on the exposure HR (shown in the second column of Table 1). Scenario 1, with no obstruction-mortality association (HR = 1.0), avoids noncollapsibility^29^ and allows quantifying the pure impact of unmeasured confounding (overestimating the exposure log(HR) by 0.057). In scenarios 2-4, the overestimation bias due to confounding is counterbalanced by a gradually increasing shift toward the null due to noncollapsibility of Cox model–based HRs, which increases with increasing true HR for obstruction exposure.^29,34^ This explains why, across scenarios 1-4, the discrepancy between the mean estimate and true log(HR) for obstruction moves from positive toward the null and then to negative (column 12 of Table 1). Interestingly, in scenarios 2 and 3, with true exposure HR of 1.3-1.5, similar to the initial estimate not adjusted for stage (HR = 1.33; 95% CI, 1.06-1.68), the impacts of confounding versus noncollapsibility almost balance each other, resulting in only very small biases of 0.019 or −0.004. Finally, scenario 5, with no confounding (obstruction-stage OR = 1.0), quantifies the expected (pure) impact of noncollapsibility (Table 1).

In additional scenarios 6 and 7, with a stronger obstruction-stage association (OR = 2.0 instead of 1.2), confounding had a much stronger impact than noncollapsibility, resulting in marked overestimation biases and low coverage rates (Table 1, columns 12 and 16). Importantly, coverage and type I error rates for scenario 6 indicate that even if true obstruction HR = 1.0, the 95% CI for the estimate not adjusted for stage will incorrectly exclude 1.0 with a 0.481 probability, as opposed to only 0.084 when assuming a weak stage-obstruction OR = 1.2 (scenario 1). Finally, scenario 8 assumed that stage had no associations with obstruction (OR = 1.0) and mortality (HR = 1.0). Then, the mean estimates, empirical SEs, and coverage rates were practically identical for oracle and imperfect datasets, with no bias for either (Table 1). This confirmed that under such assumptions, failure to adjust for stage would not affect the estimates.

## Example 2: Assessing the impact of imprecise timing of an event on its association with a time-varying exposure

### Empirical research question and data source

**Figure 2:**
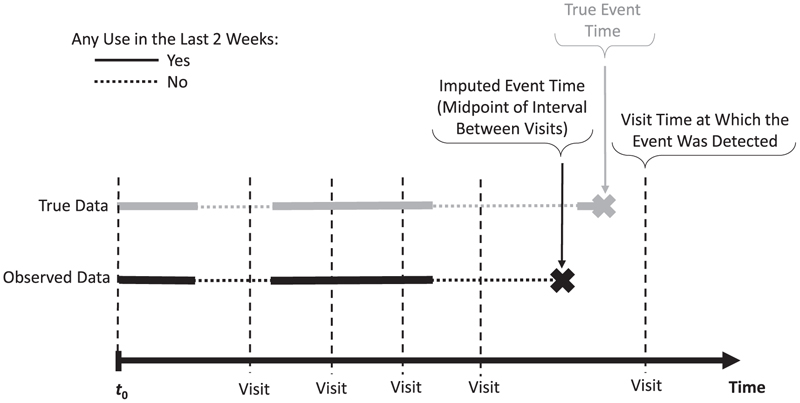
Illustration of the impact of inaccurate timing of an interval-censored event for a hypothetical study participant: the time-varying exposure metric “any use in the last 2 weeks” value differs between the true event time (exposure = yes) and the imputed event time (exposure = no) at the midpoint of the interval between the visit when the event was detected and the preceding visit.

Many cohort studies focus on endpoints that can be detected only at the discrete times of clinical assessments at medical visits. Examples include cancer recurrence, hyperlipidemia, or cognitive impairment. Then, one can only establish that the event occurred at some time during the interval between 2 adjacent clinic visits, often several months apart. Such interval-censored endpoints require specialized analytical methods.^35,36^ Yet, only a few methods were proposed to deal with associations of interval-censored events with time-varying exposures (TVEs),^37–39^ with little evidence of their use in real-world epidemiologic studies, possibly due to the lack of easily accessible software.^40^ Nonetheless, TVEs are increasingly assessed in cohort studies and are essential, for example, to account for changes over time in drug exposures in pharmacoepidemiology.^41,42^ Importantly, inaccurately timed interval-censored events may be incorrectly aligned with TVE values, as illustrated in Figure 2, where the (unknown) event time is imputed at the midpoint of the between-visits interval. This is likely to induce underestimation of the exposure-outcome association.^43^ However, the magnitude of this bias will depend on the clinic visit frequency, pattern of within-person changes in exposure, and strength of the exposure-outcome association.

To illustrate how data-driven simulations help explore the impact of interval-censoring of the outcome, we considered the association of a binary TVE representing benzodiazepine use in the past 2 weeks with a transient cognitive impairment (temporary problems with visuomotor coordination, memory, or concentration). Benzodiazepines are sedative-hypnotic drugs possibly associated with cognitive impairment.^44,45^ Using the new-users design,^46^ 1250 new elderly benzodiazepine users were followed from their first benzodiazepine prescription for up to 3 years, until one of the 285 (22.8%) events or censoring at their last follow-up visit. Daily benzodiazepine use was ascertained based on dates and durations of consecutive prescriptions.^42^ At clinic visits, on average about 3 months apart, participants reported if they experienced cognitive impairment since the last visit, resulting in an interval-censored event (see Figure 2).

Due to confidentiality restrictions, we present synthetic data similar to real-world benzodiazepine use data.^47^ This synthetic dataset, called “original” dataset below—which includes participants’ TVE (daily indicator of benzodiazepine use in the past 14 days), age, and sex—as well as dates of clinic visits, and the R scripts to reproduce the simulations are available at the link provided in the Data Availability statement.

**Step 1. Identification of the data imperfection.** The original dataset does not include the exact true times for the interval-censored cognitive impairment events.
**Step 2. Initial analyses not corrected for the imperfection.** The mean length of between-visits intervals in which the 285 events occurred was 92.2 days (interquartile range, 80-104 days). We considered two popular options for imputing times of interval-censored events: one at the midpoint of the between-visits interval or a second at the end of this interval, ie, the visit when cognitive impairment was first reported. In multivariable Cox PH models, the HR for recent benzodiazepine exposure, adjusted for age and sex, was substantially higher when event times were imputed at the midpoint (HR = 1.47; 95% CI, 1.09-2.00) than for endpoint imputation (HR = 1.21; 95% CI, 0.87-1.68).
**Step 3. Simulation assumptions.**
3a. In 4 alternative simulated scenarios, we assumed a null or increasingly strong true HR = 1.0, 1.5, 2.0, or 2.5 for the TVE (recent benzodiazepine use).
3b. We assumed that each event was equally likely to have truly occurred on any day during the corresponding between-visits interval.
**Step 4. Oracle dataset generation.** Each of the *m* = 1000 oracle datasets was generated by adapting the permutational algorithm.^20–22^ This involved 5 substeps, with substeps 4a-4b using the original data and, thus, common to all repetitions, and data-generating substeps 4c-4e repeated independently across *m* repetitions (see Appendix S4 for more details):
4a. Extract the observed daily values of the binary TVE representing benzodiazepine use in the past 2 weeks, together with sex and age, of the 1250 individuals from the original dataset.
4b. Independently, extract the times of the 285 visits **τ**_*s*_ (*s* = 1, … ,285) when the observed events were reported and each corresponding previous visit time **τ**_*s*_ − **Δ**_*s*_ from original data.
4c. Consistent with step 3b, for each event, generate the true event time *T*_*s*_ from the uniform distribution over the interval of Δ_*s*_ days [**τ**_*s*_ − **Δ**_*s*_ + 1; **τ**_*s*_], *s* = 1, … ,285.
4d. Assign each of the 285 true event times *T*_*s*_ from substep 4c to 1 individual *i, i* = 1,…,1250, defined by the vector [*X*_*i*_(*T*_*s*_), age_*i*_, sex_*i*_], where *X*_*i*_(*T*_*s*_) represents the individual’s exposure at time *T*_*s*_. The events were assigned based on the user-defined “true” multivariable PH model, based on the HR for TVE assumed in step 3a and step 2 estimates: HR = 1.066 for 1 year of age, and HR = 1.098 for male sex.
4e. Censor each of the 965 individuals not assigned an event in substep 4d at the time their follow-up ended in the original dataset.
**Step 5. Imperfect dataset generation.** For each oracle dataset simulated at step 4, we created two alternative imperfect datasets, corresponding to the two strategies for imputing event times used in step 2. First, using endpoint imputation, we imputed the event time of each participant *i*—to whom the true event time *T*_*s*_ was assigned at substep 4d—at their first clinic visit **τ**_*i*_ after *T*_*s*_. Secondly, using midpoint imputation, each event time was imputed at the midpoint between the visit times before (**τ**_*i*_ − **Δ**_*i*_) and after (**τ**_*i*_) the true event time *T*_*s*_ for individual *i* to whom *T*_*s*_ was assigned.
**Step 6. Analyses.** Simulated oracle and imperfect datasets were analyzed with multivariable Cox PH models that all included age, sex and updated TVE, but used different event times. Model 1 was fitted to oracle datasets with the exact true event times generated at substep 4c. In contrast, models 2 and 3 were fitted to imperfect datasets with times of interval-censored events imputed at, respectively, the midpoint and the endpoint of between-visits intervals during which they occurred. For all models, for each event at the (true or imputed) time *t*, we used the TVE *X*_*j*_(*t*) observed at that time *t* for all individuals *j* in the corresponding risk set. Thus, the estimation of each model relied on possibly different TVE values for the same individual.
**Step 7. Final step: summarizing results.** Results for adjusted log(HR) estimates for recent benzodiazepine use, obtained across 1000 repetitions simulated for each of the 4 scenarios with different true exposure HR, are presented in Table 2 (for models 2 and 3) and Table S3 (for model 1). As expected, the estimates are unbiased (1) for all models if there was no association (HR = 1.0), and (2) regardless of the true HR for the oracle model 1, which relies on true event times. In contrast, for all scenarios with exposure HR > 1.0, both models 2 and 3 induce systematic underestimation bias (Table 2, columns 3 and 8). This bias is, however, always higher for model 3, which imputed event times at the interval endpoint, than for model 2 based on midpoint imputation (Table 2, column 13). On the other hand, both models yielded similar empirical SE of estimates. Consequently, model 2 produced systematically more accurate estimates (ie, lower root mean squared error [RMSE]) than model 3, especially for higher exposure HRs where absolute bias is stronger (Table 2, column 14). Indeed, for HR = 2.0 or HR = 2.5, in 2/3 of simulated samples model 2 estimates are closer to the true HR than the corresponding model 3 estimates (Table 2, last column). Finally, whereas suboptimal, the coverage rate for model 2 estimates is higher in scenarios 2-4 than for model 3, which is below 50% for the strongest exposure effect (Table 2, columns 7 and 12).

Importantly, simulated scenario 1 indicates that in the absence of a true association with the TVE, model 2, fitted to imperfect data with midpoint imputation, would be unlikely to yield: (1) a 95% CI excluding the null HR = 1.0 (coverage rate of 95.7% in Table 2, implying a type I error rate of 4.3%) and (2) a point estimate exceeding the uncorrected HR = 1.47 observed in the original dataset (occurring in only 1.1% of simulated samples, data not shown). Thus, it would be very unlikely to obtain the initial midpoint estimate (HR = 1.47; 95% CI, 1.09-2.00) if there were no TVE-event association. On the other hand, in scenario 2, the 95% CI for endpoint imputation (model 3) incorrectly included HR = 1.0 in 68.6% of samples in spite of true HR = 1.5 (data not shown). Thus, the initial endpoint imputation results (HR = 1.21; 95% CI, 0.87-1.68) are also compatible with at least a moderate hazard increase associated with recent benzodiazepine use.

Overall, our simulation results also explain why the midpoint-based imputation yielded a markedly higher HR estimate in the original data, and suggest this estimate is likely less biased than the popular alternative of endpoint-based estimate, even if both likely underestimate the strength of this association.

## Additional sensitivity analyses

We then investigated whether similar results could be obtained from QBA-like sensitivity analyses of the original data. Because there is no information on when each event truly occurred within the corresponding between-visits interval, we randomly assigned each event time by sampling from the uniform distribution over this interval. In 1000 replications, we independently repeated such random redistribution of event times and refitted the Cox model, adjusting for age and sex, using the modified event times. The mean of the 1000 log(HR) estimates for the TVE was 0.310 (empirical SE, 0.089), corresponding to HR = 1.36. As this HR is similar to initial step 2 uncorrected estimates for endpoint (HR = 1.21) or midpoint (HR = 1.47) imputation, this sensitivity analysis fails to indicate the systematic, substantial underestimation bias revealed by our data-driven simulations (Table 2). These results illustrate the difficulties of relying on bias sensitivity analyses if information necessary to correct the inaccuracies in data, such as imprecise event times, is unavailable. In such situations, data-driven simulations help quantify the expected impact of the data imperfection, by comparing the estimates with the “true” parameter value, while preserving relevant features of the empirical dataset.

## Discussion

We have described steps for implementing data-driven simulations to assess the impact of study imperfections. The two examples illustrate how our approach may be applied to address various real-world challenges and yield useful insights regarding complex analytical issues. The first example permitted: (1) concluding that lacking data on cancer stage was unlikely to materially affect the estimated association of colon obstruction with survival, unless colon obstruction was strongly associated with cancer stage; and (2) quantifying the expected impacts of unmeasured confounding and noncollapsibility. The second example demonstrated that imprecise timing of a transient cognitive impairment resulted in an important underestimation of its association with a time-varying exposure (TVE) of recent benzodiazepine use. Previous research suggested that interval-censored events would likely result in bias toward the null, especially for time-invariant exposures (eg, Lindsey and Ryan^48^). However, our data-driven simulations helped estimating the expected magnitude of bias for a TVE, while accounting for both (1) frequency of visits and (2) short-term within-person changes in the TVE, both of which likely affected the bias. Furthermore, our simulation results demonstrated that estimates based on midpoint imputation are less biased in this setting.

Our data-driven simulations permit the estimation of the expected impact of particular data imperfections, in terms of discrepancies between the initial uncorrected effect estimate and the true parameter value. This helps assess the plausibility of observing specific results, in the original imperfect data, under different assumptions about the true association. For instance, for Example 1, our simulations yielded new insights about how confounding by stage could produce spurious evidence of increased mortality associated with colon obstruction, in the absence of a true association. Conversely, for Example 2, data-driven simulations demonstrated that the initial uncorrected estimates would be very unlikely if benzodiazepine use was not associated with the outcome. In this sense, our approach complements traditional probabilistic QBA methodology, described, eg, by Lash and Fink,^16^ which relies on sensitivity analyses in which the true parameter value remains unknown. Furthermore, as opposed to probabilistic QBA, our approach can be employed in settings where, to correct observed data for the relevant imperfection, one would require information not available in practice, such as the exact event times in our second example, for which our QBA-like sensitivity analyses could not reflect the important bias toward the null. On the other hand, in contrast to the alternative “QBA using generated data” approach, which relies on entirely hypothetical data,^13^ our simulated data reflect exactly several features of the real-world dataset being analyzed, which ensures direct relevance of results to this specific dataset. This is facilitated by the permutational algorithm, developed and validated for time-to-event simulations, which allows accounting for the observed distribution of event times and controlling associations with time-varying exposures.^22^ (See Appendix S5 for more detailed explanations regarding differences between our approach and existing QBA methods.)

Future research may consider some extensions of our methods and further investigation of both examples. Specifically, as our first example addresses confounding bias, it could be also discussed in terms of potential outcomes. Furthermore, our approach for choosing the bias parameters is deterministic, as in steps 3 and 4 we fix their values for each simulated scenario. However, it is possible to extend it to probabilistic simulations, with each bias parameter sampled from a prespecified distribution, similar to probabilistic QBA (eg, Lash and Fink^16^). In addition, our examples of data-driven simulations may be extended to address additional study imperfections. For Example 1, this could help assess the impact of heterogeneity related to (1) cancer severity within each category of dichotomized cancer stage at diagnosis, or (2) associations of colon obstruction and cancer stage with cancer-related vs. other causes mortality. For Example 2, simulations could be expanded to account for measurement errors in TVE, based on plausible assumptions about discrepancies between the actual use of benzodiazepines versus the use recorded in the prescription database.^41^ Our examples focus on time-to-event analyses. Yet our generic approach to data-driven simulations can be easily adapted to the analysis of other outcomes besides time-to-event. Finally, data-driven simulations could also help investigate other analytical issues arising in a given empirical dataset, beyond the impact of data imperfections. They can assess the ability of correction methods to reduce bias or compare the expected performance of alternative estimation methods, while accounting for salient characteristics of a particular real-world study.

In conclusion, we hope that our methods and results will stimulate the use of data-driven simulations in real-world epidemiologic studies, where they can complement insights offered by existing QBA methods. This can help improve analyses of observational studies, which is the goal of the STRengthening Analytical Thinking for Observational Studies (STRATOS) initiative.^49,50^

## Supplementary Material

Supplementary material is available at *American Journal of Epidemiology* online.

Supplementary Material

## Figures and Tables

**Table 1:** Simulation results for Example 1: impact of not adjusting for cancer stage on the adjusted log hazard ratio estimates for colon obstruction exposure.

Scenario	True HR for exposure [log(HR)]	True OR exposure ↔ stage	True HR for stage	Performance measures for estimated log(HR) for exposure														
				Cox model with stage (oracle data)								Cox model without stage (imperfect data)						
				Bias^a,b^	Relative bias^c^, %	Empirical SE^d^	RMSE^e^	Coverage rate 95% CI^f^	Type I error rate^g^, %	Power^g^, %		Bias^a,b^	Relative bias^c^, %	Empirical SE^d^	RMSE^e^	Coverage rate 95% CI^f^	Type I error rate^g^, %	Power^g^, %
Col. 1	Col. 2	Col. 3	Col. 4	Col. 5	Col. 6	Col. 7	Col. 8	Col. 9	Col. 10	Col. 11		Col. 12	Col. 13	Col. 14	Col. 15	Col. 16	Col. 17	Col. 18
1	1.0 [0]	1.2	4.0	0.001	N/A	0.122	0.122	0.954	4.6			0.057^b, h^	N/A	0.121	0.134	0.916	8.4^h^	
2	1.3 [0.262]	1.2	4.0	0.006	2.3	0.119	0.120	0.951		60.5		0.019^b, h^	7.1	0.122	0.123	0.939		65.5
3	1.5 [0.405]	1.2	4.0	0.002	0.6	0.117	0.117	0.950		93.1		−0.004^h^	−0.9	0.119	0.119	0.938		92.5
4	2.0 [0.693]	1.2	4.0	0.009^b^	1.3	0.111	0.112	0.952		100.0		−0.048^b^	−6.9	0.114	0.123	0.921		100.0
5	1.3 [0.262]	1.0	4.0	−0.001	−0.3	0.121	0.121	0.948		58.9		−0.040^b^	−15.1	0.121	0.127	0.939		47.0
6	1.0 [0]	2.0	4.0	0.000	N/A	0.118	0.118	0.949	5.1			0.225^b^	N/A	0.118	0.254	0.519	48.1^h^	
7	1.3 [0.262]	2.0	4.0	0.005	1.9	0.112	0.112	0.956		64.5		0.183^b^	69.8	0.116	0.217	0.643		97.4
8	1.3 [0.262]	1.0	1.0	0.002	0.9	0.120	0.120	0.944		60.7		0.002	0.8	0.120	0.120	0.945		60.5

Abbreviations: Col., column; HR, hazard ratio; N/A, not applicable; OR, odds ratio; RMSE, root mean squared error; SE, standard error.

a Mean of the 1000 estimates of log(HR) for exposure (colon obstruction) minus true log(HR) shown in column 2.

b Indicates that the 95% CI for bias excludes 0.

c Bias over true log(HR) value, presented as a percentage (not applicable if true log[HR] = 0, ie, in scenarios 1 and 6).

d Empirical SE of log(HR) estimates was calculated as the standard deviation of the 1000 log(HR) estimates.

e RMSE of the 1000 log(HR) estimates, calculated as the square root of the sum of squared bias and variance, ie, $\sqrt{\;{\text{bias}}^{2}+\text{Var}(\text{log}(\text{HR}))}$, with lower values indicating better overall accuracy of estimates.

f Proportion of the 1000 samples where the 95% CI included the true log(HR) (ideally should be very close to 0.95).

g Percentage of the 1000 samples where the 95% CI for log(HR) for exposure excluded 0. In scenarios 1 and 6, with no association (true log(HR) = 0), this corresponds to empirical type I error. In other scenarios, with a true association (true log(HR) > 0), it corresponds to empirical power.

h Indicates numbers mentioned in the text.

**Table 2:** Simulation results for Example 2: comparison of estimates for time-varying recent benzodiazepine use (exposure) from two strategies for imputing event times.

Scenario	True HR for Exposure [log(HR)]	Performance measures for estimated log(HR) for exposure														
		Model 2: event at interval midpoint						Model 3: event at interval endpoint						Comparison of model 2 and model 3		
		Bias^a,b^	Relative bias^c^, %	Empirical SE^d^	RMSE^e^	Coverage rate 95% CI^f^		Bias^a,b^	Relative bias^c^, %	Empirical SE^d^	RMSE^e^	Coverage rate 95% CI^f^		Ratio bias model 3/ model 2^g^	Ratio RMSE model 3/ model 2^h^	% repetitions model 2 closer to truth than model 3^i^, %
Col. 1	Col. 2	Col. 3	Col. 4	Col. 5	Col. 6	Col. 7		Col. 8	Col. 9	Col. 10	Col. 11	Col. 12		Col. 13	Col. 14	Col. 15
1	1.0 [0]	0.005	N/A	0.172	0.172	0.957^j^		−0.023^b^	N/A	0.177	0.178	0.967		N/A	1.03	52.2
2	1.5 [0.405]	−0.110^b^	−27.1	0.167	0.200	0.910		−0.164^b^	−40.5	0.162	0.231	0.872		1.49	1.16	57.3
3	2.0 [0.693]	−0.180^b^	-26.0	0.149	0.234	0.802		−0.257^b^	−37.0	0.152	0.298	0.649		1.43	1.27	68.3
4	2.5 [0.916]	−0.227^b^	−24.8	0.149	0.271	0.675		−0.315^b^	−34.4	0.147	0.348	0.452		1.39	1.28	71.7

Abbreviations: Col., column; HR, hazard ratio; N/A, not applicable; RMSE, root mean squared error; SE, standard error.

a Mean of the 1000 estimates of log(HR) for benzodiazepine exposure minus true log(HR) shown in column 2.

b Indicates that the 95% CI for bias excludes 0.

c Bias over true log(HR) value, presented as a percentage (not applicable if true log(HR) = 0, ie, in scenario 1).

d Empirical SE of the log(HR) estimates was calculated as the standard deviation of the 1000 log(HR) estimates.

e Root mean squared error of the 1000 log(HR) estimates, calculated as the square root of the sum of squared bias and variance, ie, $\sqrt{\;{\text{bias}}^{2}+\text{Var}(\text{log}(\text{HR}))}$, with lower values indicating better overall accuracy of estimates.

f Proportion of the 1000 samples where the 95% CI included the true log(HR) (ideally should be very close to 0.95).

g Ratio of bias for model 3 over bias for model 2, with respectively interval endpoint and midpoint event imputation.

h Ratio of RMSE for model 3 over RMSE for model 2, with respectively interval endpoint and midpoint event imputation.

i Percentage among the 1000 repetitions for which the exposure estimate from model 2 (with events imputed at midpoint of intervals) is closer to the true log(HR) (shown in column 2) than the estimate from model 3 (events imputed at endpoint of intervals).

j Indicates number mentioned in the text.

## Data Availability

All code to reproduce the simulations is available at https://github.com/mebeauchamp/DataDrivenSim-AJE2024. Data used in the two examples are publicly available, respectively, in a public R package and on the GitHub repository.
