# Supplementary material for: Data-driven simulations to assess the impact of study imperfections in time-to-event analyses
Source: Am J Epidemiol. 2024 May 6;194(1):233–42. doi: 10.1093/aje/kwae058 (PMC7617302; doi:10.1093/aje/kwae058)
Supplement: Web_Material_kwae058 [file web_material_kwae058.zip › kwae058 Abrahamowicz Supplemental Material Final.zip!/kwae058 Abrahamowicz Supplemental Material Final.pdf]

SUPPLEMENTAL MATERIAL

Data-Driven Simulations to Assess the Impact of Study Imperfections in Time-to-Event Analyses

Michal Abrahamowicz, Marie-Eve Beauchamp, Anne-Laure Boulesteix, Tim P. Morris, Willi Sauerbrei, Jay S. Kaufman, on behalf of the STRATOS Simulation Panel

CONTENTS

Table S1. Analysis results of colon cancer data available in the `survival` R package, for individuals without missing data for the variables of interest (*N* = 906 individuals, 441 deaths) 2

Table S2. Unadjusted odds ratios for pairs of variables to evaluate their correlations 3

Appendix S1. Justification of the number of simulation repetitions 4

Appendix S2. Details of the generation of cancer stage for Example 1 4

Appendix S3. Details of the permutational algorithm to generate oracle datasets for Example 1 4

Appendix S4. Details of the permutational algorithm to generate oracle datasets for Example 2 6

Table S3. Simulation results for Example 2: comparison of estimates for time-varying recent benzodiazepine use (exposure) for the model relying on true event times 7

Appendix S5. Outline of conceptual and analytical differences between our data-driven simulation approach and probabilistic quantitative bias analysis (QBA) methodology 7

Supplemental references 9

Code is available in a separate supplemental material file.

Table S1. Analysis results of colon cancer data available in the `survival` R package (1, 2), for individuals without missing data for the variables of interest (*N* = 906 individuals, 441 deaths)

**Variable**	**Level, if applicable**	**Multivariable Cox model for time to death HR (95% CI)[a]**	**Multivariable logistic regression with obstruction as dependent variable OR (95% CI)[a]**
Obstruction of colon by tumour		**1.33 (1.06, 1.68)**	--
Treatment	Observation only	1.00 (reference)	1.00 (reference)
	Levamisole	0.98 (0.78, 1.22)	0.96 (0.64, 1.45)
	Levamisole & fluorouracil	**0.70 (0.55, 0.88)**	0.86 (0.57, 1.30)
Sex	Male	1.03 (0.85, 1.25)	0.85 (0.61, 1.19)
Age (in years)		1.01 (1.00, 1.02)	**0.98 (0.97, 0.99)**
Perforation of colon		1.02 (0.60, 1.73)	**2.32 (0.98, 5.21)**
Adherence to nearby organs		1.17 (0.90, 1.51)	1.12 (0.68, 1.80)
Differentiation of tumour	Well	1.00 (reference)	1.00 (reference)
	Moderate	0.92 (0.67, 1.28)	0.84 (0.49, 1.47)
	Poor	1.33 (0.91, 1.94)	0.95 (0.50, 1.85)
Extent of local spread	Submucosa	1.00 (reference)	1.00 (reference)
	Muscle	1.29 (0.46, 3.64)	**3.09 (0.55, 58.03)**
	Serosa	**2.11 (0.78, 5.69)**	**5.48 (1.10, 99.36)**
	Contiguous structures	**3.04 (1.04, 8.85)**	**2.71 (0.38, 54.82)**
Time from surgery to registration (0 = short, 1 = long)		**1.27 (1.04, 1.56)**	1.14 (0.79, 1.65)
Having more than 4 positive lymph nodes		**2.55 (2.10, 3.10)**	0.78 (0.53, 1.15)

Abbreviations: CI, confidence interval; HR, hazard ratio; OR, odds ratio.

[a] Numbers in bold indicate significant HRs or ORs, or strong HRs or ORs with a value < 0.67 or > 1.5.

Table S2. Unadjusted odds ratios[a,b,c] for pairs of variables to evaluate their correlations

Dependent variable \ Independent variable	Level \ Level	Obstruction of colon by tumour	Treatment (reference level: observation only)		Sex	Age (in years)[d]	Perforation of colon	Adherence to nearby organs	Differentiation of tumour (reference level: well)		Extent of local spread (reference level: submucosa)			Time from surgery to registration (reference: short)
			Levamisole	Levamisole & fluorouracil	Male				Moderate	Poor	Muscle	Serosa	Contiguous structures	Long
Treatment (reference level: observation only)	Levamisole	1.03												
	Levamisole & fluorouracil	0.89	N/A											
Sex	Male	0.86	1.32	0.71										
Perforation of colon		2.55	1.19	0.86	1.01	0.91								
Adherence to nearby organs		1.16	1.15	0.84	0.88	1.11	5.11							
Differentiation of tumour (reference: well)	Moderate	0.87	0.99	0.93	1.16	1.03	0.87	0.92						
	Poor	1.11	0.81	1.18	0.98	0.93	1.15	1.54	N/A					
Extent of local spread (reference level: submucosa)	Muscle	0.58	1.06	0.88	1.21	1.07	0.30	0.77	1.22	0.64				
	Serosa	1.98	1.16	1.03	0.92	1.00	1.25	0.65	0.88	1.23	N/A			
	Contiguous structures	0.60	0.79	0.79	1.10	0.93	2.93	6.40	1.44	1.32	N/A	N/A		
Time from surgery to registration (reference: short)	Long	1.19	0.93	0.90	1.05	1.02	1.37	1.13	0.93	1.15	0.97	1.24	0.58	
Having more than 4 positive lymph nodes		0.88	1.09	0.88	0.84	0.83	0.74	0.98	0.70	2.33	0.69	1.55	1.02	0.87

[a] Numbers in red indicate strong correlations with an odds ratio < 0.67 or > 1.5.
[b] N/A is indicated when it is not applicable to calculate an odds ratio, i.e. for a pair of levels for the same variable.
[c] The upper-right part of the table is empty because correlations between the same pairs of variables are not repeated in the table.
[d] Given that age is a continuous variable, it only appears as an independent variable.

Appendix S1. Justification of the number of simulation repetitions

We relied on the approach proposed by Burton *et al.* (3) to indicate that m = 1,000 ensures an adequate ability to detect a "meaningful", i.e. moderate or strong, bias in the estimates of interest, in any models estimated at step 6 of Examples 1 and 2. To this end, we define a "meaningful" bias as the absolute difference between the mean of the m estimates and the true parameter value equal to or higher than 10% of the empirical standard error (SE) of the 1,000 estimates. This corresponds to a bias equal to a standardized difference of at least 10%, a popular criterion, e.g., for establishing covariate imbalance in causal inference. Specifically, we can calculate that m = 1,051 repetitions are sufficient to ensure 90% probability that the 95% CI for the bias will exclude 0 if the true bias corresponds to a standardized difference of 10% (see detailed calculations below). In other words, 1,000 repetitions offer approximately 90% power to detect a bias equal to 10% of the empirical SE at two-tailed α = 0.05.

Our calculations were based on equation (2) from Burton *et al.* (3), modified to account for the desired accuracy in terms of standardized difference δ^*, rather than both the accuracy and the variance of the parameter of interest:

$$m = \left(\frac{Z_{1-(\alpha/2)} + Z_{1-\theta}}{\delta^*}\right)^2$$

where $Z_{1-(\alpha/2)}$ and $Z_{1-\theta}$ are respectively the $1-(\alpha/2)$ and $1-\theta$ quantiles of the standard normal distribution to achieve $(1-\theta)$ power at the two-tailed significance level α. Specifically, we calculated to obtain 90% power to detect a bias equal to 10% of the empirical SE at two-tailed α = 0.05:

$$m = \left(\frac{1.960 + 1.282}{0.100}\right)^2 = 1051$$

Appendix S2. Details of the generation of cancer stage for Example 1

In step 4c of Example 1, a binary indicator of advanced cancer stage was simulated from a multivariable logistic model with colon obstruction, the extent of local spread and having more than four positive lymph nodes as independent variables. Based on clinical considerations and discussions with a colorectal cancer epidemiologist, we assumed that advanced cancer stage (III/IV) had a weak association with colon obstruction (odds ratio (OR) of 1.2), and was associated with the extent of local spread (ORs of 1.5, 2.0, and 2.5 for muscle, serosa, and contiguous structures, respectively, vs. submucosa) and with having more than four positive lymph nodes (OR=2.5), but not with the other covariates. The intercept of the logistic model was selected to generate an overall prevalence of advanced stage of about 35%. In sensitivity analyses the OR for the obstruction-stage association was changed to either a null or strong association (OR = 1.0 or OR = 2.0, respectively).

Appendix S3. Details of the permutational algorithm to generate oracle datasets for Example 1

We generated the oracle datasets in step 4 of Example 1 presented in the manuscript by adapting the validated permutational algorithm (4-6). This allowed us to generate event and censoring times corresponding exactly to those observed in the real-world colon cancer dataset, while reflecting also the "true" hazard ratios (HRs), assumed in step 3, for exposure and for simulated cancer stage (S_i), as well as

the HRs estimated in step 2 for all measured covariates ($\boldsymbol{X}_i$ vector for subject *i*). The specific steps of the adaptation of the permutational algorithm are the following:

(4a) Extract 906 individual $\boldsymbol{X}_i$ vectors (i = 1,…,906) of observed values of the binary indicator of colon obstruction exposure and all measured covariates, preserving all their relationships.

(4b) Independently, extract all 906 observed real-world outcomes: 441 times of death and 465 (906-441) censoring times. For each outcome i =1,…,906, the tuple (t_i^*, δ_i^*) indicates the follow-up time t_i^* and the corresponding outcome status δ_i^* (1 for event, 0 for censoring). Order the tuples by increasing time t_i^*.

(4c) Generate a binary indicator of higher cancer stage S_i for each patient, i = 1,…,906, from the binomial distribution, with a probability calculated using a multivariable logistic model based on patient-specific $\boldsymbol{X}_i$ vectors from sub-step 4a and corresponding ORs assumed in step 3b (see Appendix S2 for details).

(4d) Starting from the earliest observed outcome time t_1^*, randomly assign each of the outcome tuples (t_i^*, δ_i^*), i = 1,…,906, to an individual k defined by the vector of exposure/covariate values and generated cancer stage ($\boldsymbol{X}_k$, S_k).

- If δ_i^* = 1, t_i^* represents an event time and the individual selected for this event time is sampled with a probability based on the user-defined "true" multivariable PH model, with HRs for colon obstruction and cancer stage based on step 3 assumptions (see the manuscript), and HRs for measured covariates equal to the adjusted estimates from step 2. Accordingly, the probability that event time t_i^* is assigned to individual k is defined by

$$P_{k,t_i^*} = \frac{\exp\left[\boldsymbol{\beta}'_X \boldsymbol{X}_k(t_i^*) \;+\; \log(4.0)\, S_k(t_i^*)\right]}{\sum_{j \in R_i} \exp\left[\boldsymbol{\beta}'_X \boldsymbol{X}_j(t_i^*) \;+\; \log(4.0)\, S_j(t_i^*)\right]}$$

  where $\boldsymbol{\beta}_X$ is the vector of log HRs for obstruction and measured covariates: HR for obstruction was either 1.0, 1.2, 1.5, or 2.0, depending on the simulation scenario; and log HRs for measured covariates are shown in the 3rd column of Table S1. Log HR for cancer stage S_k was set to log(4.0). The risk set R_i for event time t_i^* was defined as the set of individuals that have not been assigned yet an outcome (event or censoring) time. Remove the selected individual from risk sets for all outcome times to be subsequently assigned after t_i^*.

- If δ_i^* = 0, t_i^* represents a censoring time and is assigned by simple random sampling to an individual still at risk in the risk set R_i, i.e. with a probability $p_i = 1/\text{size}(R_i)$. Remove the selected individual from risk sets for all outcome times to be subsequently assigned after t_i^*.

This adaptation of the permutational algorithm can be implemented using the `permalgorithm` function from the `PermAlgo` package in R (7), which corresponds to the description of the algorithm presented by Sylvestre and Abrahamowicz (6), with the difference that in the current simulation study we used the *observed* event and censoring times and *observed* exposure and covariate values from our real-word dataset, instead of generating them (6). See the R scripts to reproduce our simulations for Example 1, available at https://github.com/mebeauchamp/DataDrivenSim-AJE2024, for details about how to use the `permalgorithm` function (7) in the context of a simulation study based on a real-word dataset with *time-invariant* covariates.

Appendix S4. Details of the permutational algorithm to generate oracle datasets for Example 2

For the generation of each oracle dataset (step 4) for Example 2 presented in the manuscript, the specific steps of the adaptation of the permutational algorithm are the following:

(4a) Extract the observed daily values of the binary time-varying exposure (TVE) representing benzodiazepine use in the past two weeks, together with sex and age, of the 1,250 individuals from the original dataset.

(4b) Independently, extract the times of the 285 visits τ_s (*s* = 1,…,285) when the observed events were reported and each corresponding previous visit time τ_s - Δ_s from original data.

(4c) Consistent with step 3b described in the manuscript, for each event, generate the true event time T_s from the uniform distribution over the interval of Δ_s days [τ_s - Δ_s + 1; τ_s], *s* = 1,…,285. Order all T_s by increasing value.

(4d) Starting from the earliest "true" event time T_1, randomly assign each event time T_s, *s* = 1,…,285, from sub-step 4c to one individual *i*, *i* = 1,…,1250. The probability that an individual was selected for an event was based on the user-defined "true" multivariable PH model, where for each simulated scenario we used the HR for TVE assumed in step 3a (see the manuscript) and HRs for 1 year increase of age and for male sex were fixed to 1.066 and 1.098, based on the corresponding step 2 estimates. Accordingly, the probability that individual *k* is selected for event time T_s is defined by

$$P_{k,T_s} = \frac{\exp\left[\beta_X X_k(T_s) \ + \ \log(1.066)\ age_k + \log(1.098)\ sex_k\right]}{\sum_{j \in R_s} \exp\left[\beta_X X_j(T_s) \ + \ \log(1.066)\ age_j + \log(1.098)\ sex_j\right]}$$

where $X_k(T_s)$ is the TVE value for individual *k* at time T_s; age_k and sex_k are individual *k*'s covariate values; and β_X is the TVE true log HR (HR=1.0, 1.5, 2.0 or 2.5 depending on the simulation scenario). The risk set R_s for event time T_s was defined as the set of individuals: a) for whom the TVE $X_k(t)$ value was known up to event time T_s in the original dataset, and b) who have not been already selected yet for any earlier event at $t \leq T_s$. Remove the individual selected for event time T_s from risk sets for all subsequent event times.

(4e) Censor each of the 965 individuals *not* assigned an event in the previous step 4d at the time when their observed follow-up ended in the original dataset (regardless of the status observed in the original data for this individual).

Once sub-steps 4a-4e are completed, stop the follow-up of the 1,250 individuals at their assigned event or censoring times for this generated oracle dataset. This implies deleting, for each individual assigned an event, all rows in the original dataset corresponding to times later than their assigned event time.

Note that this adaptation of the permutational algorithm above *cannot* be implemented with the `permalgorithm` function available in the `PermAlgo` package in R (7). Indeed, the `permalgorithm` function requires knowing the exposure and covariates values, for all subjects, up to the maximum length of follow-up, in such a way that any subjects can be assigned any event/censoring times. However, in our original dataset, for each individual the updated values of the binary time-dependent indicator of benzodiazepine use are *unknown* after the end of their follow-up time, which corresponds to either the clinic visit at which an event was reported or their last clinic visit during follow-up. Therefore, in a

simulated dataset, event times cannot be assigned after the end of follow-up of individuals in the original dataset. (Note that, in contrast, we were able to use the `permalgorithm` function (7) in Example 1 of the manuscript because all variables were *time-invariant* and, thus, their values are constant and then known for all individuals for the maximum length of follow-up.) Instead, a new function (`permalgorithm.realdat`) was created to adapt the permutational algorithm to the context of real-word time-dependent data where individual exposure and/or covariates values are known only up to a specific time that differs across individuals. The code for the version of the function used for our simulations is available at https://github.com/mebeauchamp/DataDrivenSim-AJE2024. For the latest version of this function, see the function `permalgorithm.realdat` that will be implemented in the next version of the `PermAlgo` package (7).

Table S3. Simulation results for Example 2: comparison of estimates for time-varying recent benzodiazepine use (exposure) for the model relying on true event times

**Scenario**	**True HR for Exposure [log(HR)]**	**Performance Measures for Estimated log(HR) for Exposure**				
		**Model 1: True Event Times**				
		**Bias[a,b]**	**Relative Bias[c], %**	**Empirical SE [d]**	**RMSE[e]**	**Coverage Rate 95% CI[f]**
1	1.0 [0]	-0.009	N/A	0.180	0.180	0.955
2	1.5 [0.405]	-0.004	-1.0	0.166	0.166	0.949
3	2.0 [0.693]	0.000	-0.1	0.146	0.146	0.959
4	2.5 [0.916]	0.002	0.2	0.140	0.140	0.949

Abbreviations: CI, confidence interval; HR, hazard ratio; N/A, not applicable; RMSE, root mean squared error; SE, standard error.

[a] Mean of the 1,000 estimates of log(HR) for benzodiazepine exposure minus true log(HR) shown in column 2.

[b] Indicates that the 95% CI for bias excludes 0 (none in this table).

[c] Bias over true log(HR) value, presented as a percentage (does *not* apply if true log(HR)=0, i.e. in scenario 1).

[d] Empirical SE of the log(HR) estimates was calculated as the standard deviation of the 1,000 log(HR) estimates.

[e] Root mean squared error of the 1,000 log(HR) estimates, calculated as the square root of the sum of squared bias and variance, i.e. $\sqrt{\text{bias}^2 + \text{Var}(\log(\text{HR}))}$, with lower values indicating better overall accuracy of the estimates.

[f] Proportion of the 1,000 samples where the 95% CI included the true log(HR) (ideally should be very close to 0.95).

Appendix S5. Outline of conceptual and analytical differences between our data-driven simulation approach and probabilistic quantitative bias analysis (QBA) methodology

First, we believe that our data-driven simulation approach *complements* traditional probabilistic QBA methodology, described e.g. in the seminal paper by Lash and Fink (8). In a nutshell, the main difference between the traditional probabilistic QBA *versus* our approach is that the latter allows a direct estimation of the expected impact of given data imperfections, in terms of the expected discrepancies between initial

effect estimates (*not corrected* for the imperfections) and the true value of the corresponding parameter. In contrast, most published QBA analyses use sophisticated probabilistic *sensitivity analyses* in which the true value of the parameter for the association of interest remains *unknown*. In these QBA analyses, the main objective is to explore how the effect estimates of interest, obtained in a specific real-world study, may change if the particular data imperfections were corrected for. To this end, based on literature and/or substantive knowledge, the analysts first specify assumptions regarding the plausible values of the relevant bias parameters, and then use Monte Carlo simulation methods to create multiple corrected versions of the original observed imperfect data. Comparison of estimates obtained from the corrected datasets vs. the original dataset provides an empirical assessment of the impact of the imperfections of interest (e.g., (8)). Equally important, if the bias parameters are selected based on (even approximately) valid assumptions, the QBA-corrected estimates are expected to be more accurate than the original uncorrected estimates. However, similar to other sensitivity analyses of observed data, these traditional QBA analyses do *not* allow the analysts to directly quantify the impact of the data imperfection on, e.g., bias, overall accuracy (in terms of root mean squared error (RMSE)), and coverage rate of confidence intervals, as all these performance measures would require knowledge of the true value of the parameter of interest. Thus, traditional QBA sensitivity analyses do not permit to quantify the differences in, e.g., bias or RMSE between uncorrected *vs.* QBA-corrected estimates. In this sense, by providing direct estimates of all the above performance measures and comparing their values for imperfect vs. oracle datasets (as illustrated in Tables 1 and 2 of the manuscript and Table S3), the results of our data-driven simulations provide insights that are *complementary* to those offered by the traditional probabilistic QBA analyses.

From this perspective, our data-driven simulations are more similar to an alternative, arguably less often employed, QBA approach identified in a recent review/tutorial of QBA methods (9) as "QBA using generated data". This approach relies on simulated data to explore the expected magnitude of bias induced under specific *hypothetical* scenarios. Even so, an important difference between generated data-based QBA *versus* our data-driven simulations is that the former uses entirely simulated, *hypothetical* data, whereas our simulations reproduce exactly several salient features of the specific real-world dataset being analyzed. In particular, we ensure that, in all simulated samples, the distributions of (a) (possibly time-varying) exposure, (b) all relevant measured covariates, and (c) time-to-event outcomes (event times) are all identical to the corresponding distributions observed in the real-world dataset. This is relevant as, jointly, these distributions may affect both the point estimate of the HR for exposure and its precision. These aspects of our approach are more similar, in spirit, to "traditional probabilistic QBA", as they make our simulation results directly relevant for the specific real-world dataset. Thus, our data-driven simulation approach attempts to <u>*combine*</u> some aspects of both existing QBA approaches. (i) On one hand, similar to traditional probabilistic QBA sensitivity analyses, it permits assessing the expected impact of relevant data imperfection(s) on the effect estimates obtained in a specific real-world study. (ii) On the other hand, similar to "QBA using generated data", it allows contrasting the imperfect real-world estimates against the known true parameter values.

There is also a more technical difference between our approach *versus* the existing traditional probabilistic QBA sensitivity analyses, which is practically important for some settings. Specifically, in some studies it will be difficult, or even impossible, to apply the QBA sensitivity analyses to correct the observed real-world data for a particular imperfection, as this would require knowledge that, by its nature, cannot be available (even approximately) to the researchers. In the main manuscript, we illustrate the resulting difficulties in the context of our Example 2, which focuses on unknown times of the occurrence of interval-censored events. In particular, in the sub-section on "Additional sensitivity analyses", at the end of step 7 for Example 2, we show that sensitivity analyses based on random re-distribution of

(unknown) event times fail to reflect a systematic, substantial (34%-41%) bias toward the null demonstrated by the results of our data-driven simulations in Table 2. Finally, we note that to carry out data-driven simulations for this example, it was essential to rely on our permutational algorithm which ensures that the resulting, partly simulated, time-to-event data are consistent with the pre-specified true hazard ratio for the time-varying exposure.

Supplemental references

1. Therneau TM. A Package for Survival Analysis in R. R package, version 3.2-13: https://CRAN.R-project.org/package=survival; 2021.
2. Moertel CG, Fleming TR, Macdonald JS, et al. Fluorouracil plus levamisole as effective adjuvant therapy after resection of stage III colon carcinoma: a final report. *Ann Intern Med* 1995;122:321-326.
3. Burton A, Altman DG, Royston P, et al. The design of simulation studies in medical statistics. *Stat Med* 2006;25:4279-4292.
4. Abrahamowicz M, MacKenzie T, Esdaile JM. Time-dependent hazard ratio: modeling and hypothesis testing with application in lupus nephritis. *J Am Stat Assoc* 1996;91:1432-1439.
5. MacKenzie T, Abrahamowicz M. Marginal and hazard ratio specific random data generation: applications to semi-parametric bootstrapping. *Stat Comput* 2002;12:245-252.
6. Sylvestre MP, Abrahamowicz M. Comparison of algorithms to generate event times conditional on time-dependent covariates. *Stat Med* 2008;27:2618-2634.
7. Sylvestre MP, Evans T, MacKenzie T, et al. PermAlgo: Permutational algorithm to generate event times conditional on a covariate matrix including time-dependent covariates. R package, version 1.2: https://CRAN.R-project.org/package=PermAlgo; 2022.
8. Lash TL, Fink AK. Semi-automated sensitivity analysis to assess systematic errors in observational data. *Epidemiology* 2003;14:451-458.
9. Banack HR, Hayes-Larson E, Mayeda ER. Monte carlo simulation approaches for quantitative bias analysis: a tutorial. *Epidemiol Rev* 2021;43:106-117.
